# Experimental Study on the Influence of Wire-Saw Wear on Cutting Force and Silicon Wafer Surface

**DOI:** 10.3390/ma16103619

**Published:** 2023-05-09

**Authors:** Lie Liang, Shujuan Li, Kehao Lan, Ruijiang Yu, Jiabin Wang, Wen Zhao

**Affiliations:** School of Mechanical and Precision Instrument Engineering, Xi’an University of Technology, Xi’an 710048, China

**Keywords:** wire saw, wear, cutting force, surface roughness, surface profile, failure mode

## Abstract

Hard and brittle materials such as monocrystalline silicon still occupy an important position in the semiconductor industry, but hard and brittle materials are difficult to process because of their physical properties. Fixed-diamond abrasive wire-saw cutting is the most widely used method for slicing hard and brittle materials. The diamond abrasive particles on the wire saw wear to a certain extent, which affects the cutting force and wafer surface quality in the cutting process. In this experiment, keeping all the given parameters unchanged, a square silicon ingot is cut repeatedly with a consolidated diamond abrasive wire saw until the wire saw breaks. The experimental results show that the cutting force decreases with the increase in cutting times in the stable grinding stage. The wear of abrasive particles starts at the edges and corners, and the macro failure mode of the wire saw is fatigue fracture. The fluctuation of the wafer surface profile gradually decreases. The surface roughness of wafer is steady during the wear steady stage, and the large damage pits on the wafer surface are reduced in the whole process of cutting.

## 1. Introduction

Monocrystalline silicon is a good semiconductor material with good thermal stability, small linear coefficient of thermal expansion, and higher melting temperature for wider process tolerance [1,2]. It is easy to prepare large-sized crystals, and its purity is easy to achieve and control. It has an irreplaceable position in the industry, accounting for 90% of the entire semiconductor material market [3]. Meanwhile, due to the high strength and hardness of monocrystalline silicon, processing technology has always been a hot research topic [4]. The slicing of monocrystalline silicon is the primary process of processing [5], accounting for about 30% of the total cost of the entire process [6,7]. Diamond-wire-saw cutting is widely used in the cutting process of hard and brittle materials [8,9,10] due to its advantages such as minimal material waste, low cutting force, and narrow saw gaps [11,12,13]. During the wire-saw machining process, process parameters such as part feed rate and wire velocity have an impact on the cutting process [13]. In addition, the abrasive particles on the wire inevitably wear during the cutting process, and the impact of wear on the cutting force and wafer quality during the wafer machining process also needs to be considered.

The quality of the wafer is the evaluation standard of processing technology. The real-time quality measurement cannot be carried out in the machining process. Many studies have found a close relationship between cutting force and wafer quality during the cutting process [14]. Cutting force is the interaction force between the wire saw and the part, which can be detected in real time, so most researchers have studied the changes of cutting force in many aspects. Liu et al. [15] developed a model to calculate the cutting depth and cutting forces in view of the indentation fracture mechanics, and the effect of process parameters on abrasives cutting behavior was studied. The random distribution characteristic of abrasives and the elasticity of resin layer were taken into account in this model. Li et al. [1] derived an analytical cutting force model for the wire saw machining process considering the change of the contact length, based on analyzing the forces generated from the chip formation and friction of a single abrasive. Wang et al. [14] pointed out that the cutting forces are key factors affecting the surface quality and sub-surface quality of wafers. Therefore, the cutting force prediction method considering cutting process parameters was established, which combines the brittle fracture removal and ductile removal of single abrasives. Moreover, the influence of lateral cracks on material removal method and the friction component in the cutting force calculation of single abrasives are considered in the study. Hui et al. [16] studied the relationship between the tangential cutting forces and wire velocities, feed rates, and the material removal rates, and then found that the tangential cutting forces increased with the decrease in wire velocities and the increase in feed rates and were in a good linear relationship with the material removal rates. Wang et al. [17] established a single-abrasive force model. In the process of cutting force research, it could be found that there was a close relationship between cutting depth and cutting force. The purpose of studying the cutting force was to clarify the cutting mechanism, optimize the cutting parameters, and improve the wafer quality after cutting. Tang et al. [18] used the irregular polyhedron generated by the random space plane ball method to simplify the abrasive particles and used the random algorithm to establish the spatial coordinates of the abrasive particle center. A more realistic 3D model of the wire-saw surface was established. The cutting force simulation model of a multi-abrasive wire saw cutting hard and brittle materials was established by the finite element method. Wallburg Florian et al. [19] analyzed and assumed that there was a relationship between the force and the material removal rate. The finite element model was used to calculate the material removal coefficient. Zhiyuan Lai et al. [20] found that the contact state between the part and the wire, the cutting force, and the material removal were three factors that interacted with each other. The research on cutting force is basically divided into two aspects: one is the derivation of its analytical formula, and the other is the influence of machining parameters on cutting force. The machining parameters here are set and controlled in the experiment, and there is little research on the impact of abrasive wear changes on the cutting force.

Now there are many studies on the parameters in the machining process: part feed rate, wire saw velocity, wire tension, and so on. In the actual machining process, when cutting monocrystalline silicon with a wire saw, diamond abrasive particles are worn to a certain extent, and abrasive wear also has an impact on the machining process. Kumar and Kaminski et al. [21] reported that the wafers exhibit greater evidence of ductile removal, lower surface roughness, fewer but slightly deeper subsurface cracks, and lower average fracture strength with increased wire wear. Uygar Pala et al. [7] observed the main abrasive grain wear modes were blunting and rounding of the diamond grains and wear modes were expected to lead to an increase in cutting forces. Knoblauch and Boing et al. [22] investigated the wire wear started with the removal of the nickel matrix covering the grains, and the nickel matrix was then plastically deformed and piled up at the sides of the grains, contributing to their support during the cutting. Gupta et al. [23] observed with increased wire wear that the wafers showed lower roughness and greater evidence of ductile removal, and subsurface cracks were more numerous and slightly deeper for wafers. Wang et al. [24] studied the wear characteristics of diamond and found that the main wear mechanism attributed to diamond segments wear was the fracture and falling of diamond particles caused by heavy loads, especially in rocking reciprocating sawing mode, and the average protrusion height of diamond particles was related to loads and the bonding strength of the matrix. Haiyong Wu [25] measured and tracked the wear amount, wear morphology, and scratch force of single-diamond abrasive grain and established stress models of different contact forms. The results showed that the abrasive particles in the form of surface contact could remove the most materials and had the best wear resistance. Yihe Liu et al. [26] studied the influence of the evolution of saw wire cutting performance on the surface roughness and topography of Si3N4 ceramic sawed chips by analyzing the changes of saw wire wear topography, fracture force, bow angle, and kerf loss during the sawing process. The results showed that the surface roughness along the wire motion direction and the part feed direction decreased first and then increased with the change of wire cutting performance. Yan Wang et al. [5] pointed out that abrasive wear had a great impact on cutting force. According to the law of abrasive wear on the wire saw, the finite element model of the wire saw under different abrasive wear conditions was established.

The above studies show that diamond abrasive wear has an impact on the cutting process and the surface quality of the wafer after cutting. Abrasive wear also affects the change of cutting force in the cutting process. However, in current research, some researchers focus on single abrasive particles for wear research, which cannot be directly reflected in the machining process, or observe the cutting force change by cutting a small number of wafer chips, without a complete display of the entire wire saw usage process. In this paper, multiple wafers will be cut while keeping other processing parameters unchanged. During the cutting process, abrasive wear becomes a variable that affects the results. The wire saw cuts wafers until the wire breaks, causing a longer wear process of the abrasive particles. The changes in cutting force and wafer quality were observed, indicating the impact of abrasive wear on the cutting process.

## 2. Experiment Setup

**Figure 1:**
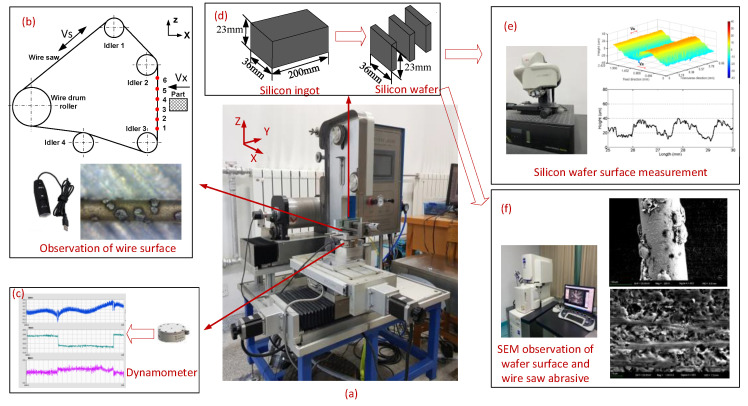
Schematic diagram of monocrystalline silicon cutting and testing methods. (**a**) The wire saw machine. (**b**) Observation of wire surface. (**c**) Dynamometer and force acquisition interface. (**d**) Schematic diagram of silicon ingot and silicon wafer. (**e**) Silicon wafer surface measurement. (**f**) SEM observation of wafer surface and abrasives.

The wire saw machine used for the experimental studies conducted in this paper is shown in Figure 1a. A wire drum roller, three idler pulleys, and an adjustment pulley form the wire feed mechanism. The part moves in the X and Y directions via two linear axes driven by stepper motors. The wire matrix is nickel plated and impregnated with JR2-type diamond abrasives having an average grain size of 50–60 μm. In the experiment, commonly used experimental parameters are shown in Table 1, the wire saw velocity Vs is 1 m/s (the commonly used wire velocity range for slow cutting is 0.8–1.6 m/s), and the part feed rate Vx is 0.75 mm/min (the commonly used part feed rate range is 0.5–1 mm/min). The part is silicon monocrystal (Si), and the cutting machine is a small-sized cutting machine. During the cutting process of circular silicon ingots, the contact length between the part and the wire is always changing, and the rectangular part can effectively avoid this. In order to reduce variables during the cutting process, the rectangular part is selected for the experiment. The part size is 36 mm × 23 mm × 200 mm, and the cutting surface size is 36 mm × 23 mm, as shown in Figure 1d. After cutting a wafer, wafer cutting is repeated until the wire saw breaks. During the cutting process, the set parameters are kept unchanged.

Fixed-diamond abrasive wire-saw cutting involves fixing many diamond abrasive particles on the wire. After the wire saw is tensioned on the machine tool, the part moves in the direction of the wire saw, and the wire saw moves on the cutting surface as shown in Figure 1b. By interacting between the wire saw and the part, the abrasive particles on the wire saw press into the part, and as the wire moves, the part material is removed, forming a cutting process. The cutting force is collected in real-time throughout the entire machining process, the abrasive particles’ state is observed after cutting, and the wafer quality is evaluated.

The dynamometer is consolidated with the part to measure the forces in the cutting process in real time in Figure 1c. The dynamometer is an ATI FT19500, with resolution of 1/160–1/160–1/80 and measurement range of 32 N–32 N–100 N in the X–Y–Z direction. After each wafer is cut, the wire between idlers on both sides of the part is measured by selecting 6 position points at an equal distance as shown in Figure 1b. The six positions of the wire saw are photographed by Anyty 3R-MSUSB601 to observe the surface of the wire saw. After the silicon ingot is cut into silicon wafers, as shown in Figure 1d, different measurements are made on the surface of silicon wafers. Leica DCM3D white light confocal interference microscope is used to measure wafer surface roughness and surface profile in Figure 1e. SEM of Merlin Compact takes photos of the wafer surface and wire saw to observe the scratches on the wafer surface and the wear of abrasives as shown in Figure 1f. 

## 3. Experiment Results and Discussion

Using the equipment in Figure 1a to cut the silicon ingot, 15 wafers were cut completely when the wire saw broke. During the cutting process, the degree of abrasive particle wear changes. If other set parameters remain unchanged, the changes in cutting force and wafer surface during the cutting process are caused by abrasive wear.

### 3.1. Analysis on Variation of Cutting Forces

**Figure 2:**
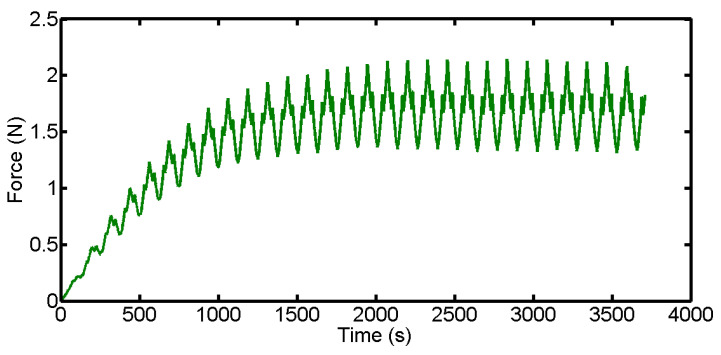
Variation of normal cutting forces for a wafer.

Figure 2 shows the cutting force of the whole process collected in real time when cutting a wafer. When wire saw cuts square monocrystalline silicon, there are two stages of normal cutting force in each cutting. The first stage is that the cutting force increases rapidly from 0 N with cutting. When the removal amount is equal to the amount of the part feed, the cutting force is in a stable state.

**Figure 3:**
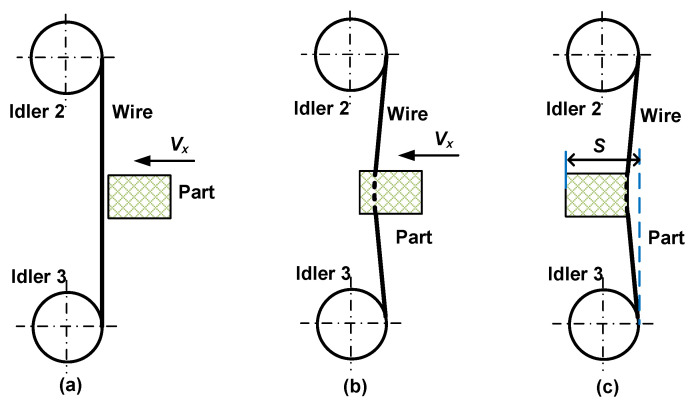
The positional relationship between the wire and the part before and during processing. (**a**) No cutting. (**b**) Cutting in progress. (**c**) Cutting is about to end.

Before cutting, the wire saw does not contact the part, as shown in Figure 3a, and the cutting force is 0 N. As the part is fed, it contacts the wire saw, forming a bow angle, as shown in Figure 3b. The situation of wire bending continues until the end of cutting. The actual movement distance of the part during the cutting process is *S* in Figure 3c, which is larger than length of the part.

**Figure 4:**
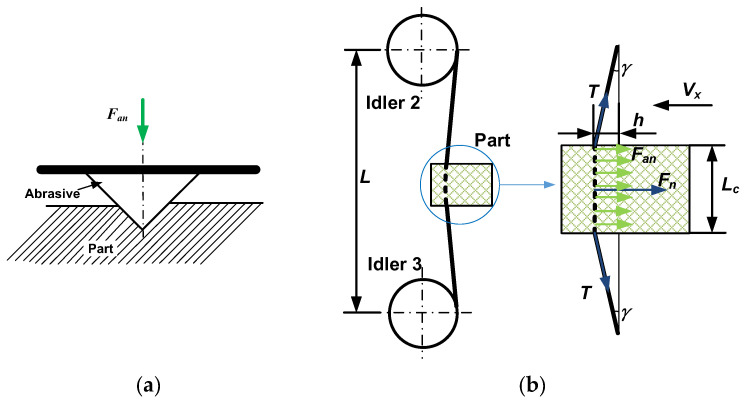
Schematic diagram of the forces on the abrasive and the part during cutting. (**a**) Positive force on single abrasive. (**b**) The force on the part.

According to the research [9], the depth of abrasive particles pressed into the part is related to the positive force *F_an_* (the force applied to a single abrasive particle in the direction of pressing into the part as shown in Figure 4a) in Equation (1). The abrasives are pressed into the part, and the material is removed as the wire saw moves. The cutting force is related to the wire tension in Figure 4b. The cutting force is the component of the wire tension in the feeding direction of the part, and the relationship is shown in Equation (2). *L* and *L_C_* are determined values in the experiment, and the bow angle *γ* changes with the bending distance of the wire in Equation (3). At the initial stage, the bow angle *γ* is relatively small, and the normal upward component of the wire tension on the part is relatively small. The amount of material fed (the volume of material that should be removed corresponding to the part feed per unit time) is greater than the amount of material removed by abrasive particles (the actual volume of material removed per unit time). With the part feeding and the accumulation of unremoved materials, the bow angle γ becomes larger, and the component of tension in the part feed direction also increases. This forms the first stage of cutting force growth.

(1) $$F_{an}=\frac{\pi }{2}{\left(g\tan\left(\theta \right)\right)}^{2}H$$

(2) $$F_{n}=2T\sin(\gamma )$$

(3) $$\gamma =\arctan\frac{2h}{\left(L-L_{c}\right)}$$

where *F_an_* is the positive force on the single abrasive, as shown in Figure 4a; g is the depth of penetration of the abrasive, 2*θ* is the abrasive tip angle, and *H* is the hardness of the part. *F_n_* is the cutting force on the part (combined force of positive force of abrasives), *T* is the wire tension, *γ* is the wire bow, *L* is the distance between the centers of idlers 2 and 3, L*_c_* is the thickness of the part, and *h* is the bending distance of the wire in Figure 4b. 

With the increase in cutting force, the positive force on a single abrasive grain also increases so that the depth of penetration of the abrasive becomes larger and the material removal amount increases. Zhiteng Xu et al. [27] explains this view in their research on the bow angle of wire saw in the cutting process. When the amount of material removed is equal to the amount of the part feed, the cutting process enters a stable stage. The bow angle and cutting force are stable near a certain value; that is, the average cutting force does not change until a wafer is cut off.

**Figure 5:**
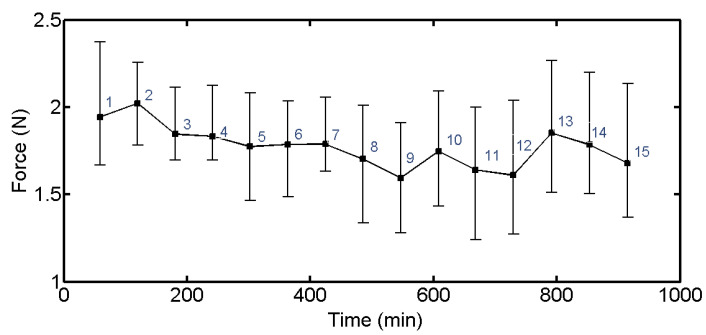
Variation of normal cutting forces for 1–15 wafers.

During the repeated cutting of 15 wafers, the setting parameters such as wire saw velocity and part feed rate do not change, but the wear degree of abrasive particles changes during the cutting process. In order to study the change of cutting force with the number of cuts, the cutting forces of 15 wafers at the stable stage are compared in Figure 5. 

The cutting force in the second slicing process is greater than that in the first slicing process in Figure 5. The cutting force decreases gradually from the second to the ninth and fluctuates from the tenth to the fifteenth. This is because the abrasive state is unstable at the initial stage of cutting. During the process of cutting the first wafer, there are some large abrasive particles that remove the material irregularly, and the removal amount is large. At the same time, the interaction between abrasive particles and the part results in the wear of large abrasive particles. The cutting of the first wafer is in the running-in stage between abrasive particles and part, which is also the initial stage of abrasive wear. 

**Figure 6:**
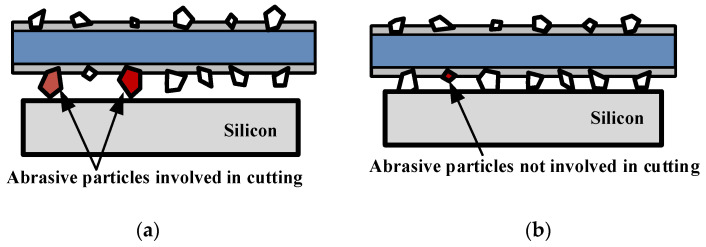
Schematic diagram of abrasive particles involved in cutting. (**a**) The abrasives state in the early of cutting. (**b**) The abrasives state in the middle of cutting.

The second to ninth wafers are in the stable wear stage. Large abrasive particles are slowly ground down, and some new abrasive particles participate in cutting process, as shown in Figure 6. According to Equation (2), the component of wire saw tension in the direction of normal cutting force depends on the bow angle between wire saw and part. The bow angle is formed due to the accumulation of unremoved material, which is caused by the material removal amount being less than the part feed. The part feed rate is determined and does not change. The amount of material removal is related to abrasive particles. As the removal amount of abrasive particles increases, the bending degree of wire saw decreases, so the component of wire saw tension in the direction of normal cutting force decreases.

When cutting to the 10th and 13th wafers, the cutting force increases, but between the 10th and 12th wafers and between the 13th and 15th wafers, the cutting force decreases. The number of abrasive particles falling off and losing cutting ability increase, resulting in the reduction of the number of abrasive particles involved in cutting so that the bow angle increases under the effect of material accumulation, and the cutting force increases. After a part of the abrasive particles fall off, the cutting enters a short stable wear stage of abrasive particles, and part of the abrasive particles fall off again with the cutting. This formed the situation of the 10th to 15th wafers in the later stage of processing.

### 3.2. Abrasive Wear Analysis

In the experiment, the parameters such as part feed rate and wire saw velocity are stable during cutting. However, the randomly distributed abrasive particles on the wire saw interact with the part. The abrasive particles remove the material, and the part wears the abrasive particles accordingly.

**Figure 7:**
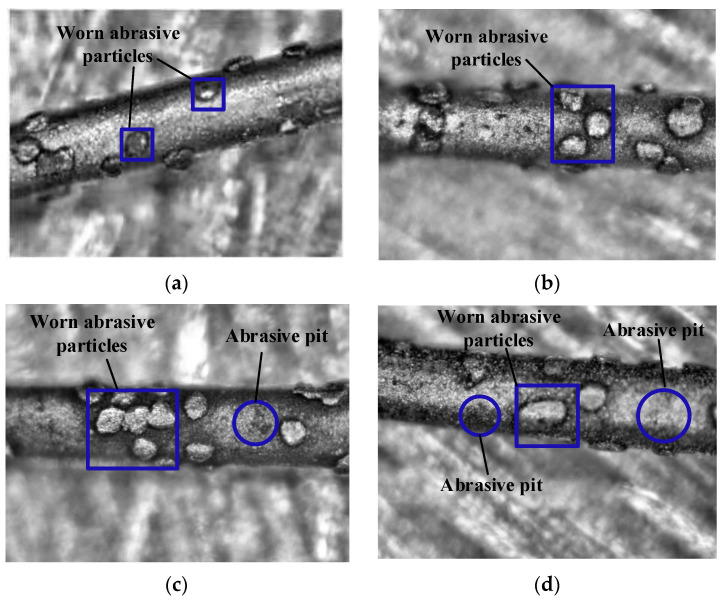

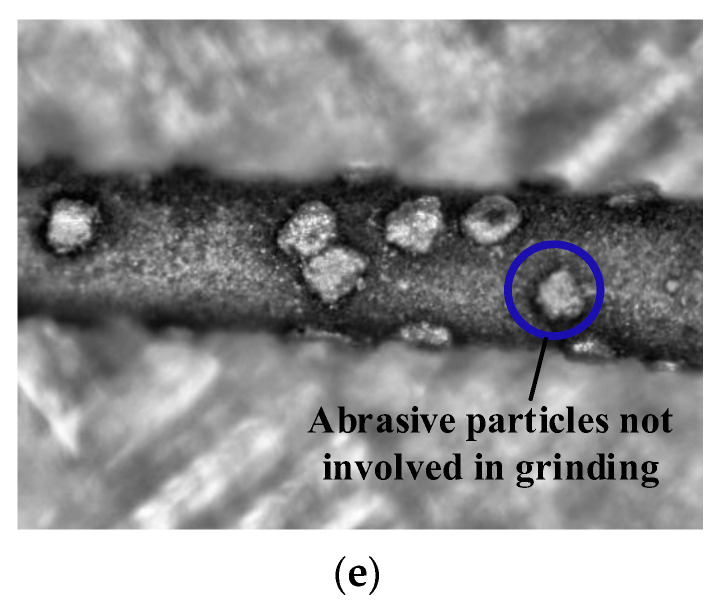
Enlarged photos of saw wire during the experiment. (**a**) Wire photo after cutting the first wafer. (**b**) Wire photo after cutting the fifth wafer. (**c**) Wire photo after cutting the 10th wafer. (**d**) Wire photo after cutting the 15th wafer. (**e**) Wire photo after cutting the 15th wafer.

During the experiment, the wire will be photographed after each wafer is cut. Figure 7 shows the wire photos after the cutting of the 1st, 5th, 10th, and 15th wafers. After cutting the first wafer, the edges of some abrasive grains become bright, as shown in Figure 7a. Among the abrasive grains in the figure, only one or two of the abrasive grains’ edges have been ground. It shows that in the process of cutting the first wafer, the number of abrasive particles involved in cutting is small, and the initial grinding process is to remove the material by the abrasive particles’ edges. It can be seen from Figure 7b,c that the bright area of the abrasive grains is increasing, not only the number of abrasive grains but also the grinding area of the abrasive grains themselves. When the 10th wafer is cut, most of the abrasive grains have been involved in cutting, and the surfaces of most abrasive grains involved in cutting become smooth, indicating that abrasive grains’ surfaces are involved in processing, not just the edges. When cutting the 10th wafer, the pit marks of abrasive particles falling can be observed. Section 3.1 in the paper determines that the abrasive particles in the stable grinding stage fall more when cutting the 10th piece, which is corresponding. Figure 7d,e are wire photos of different measurement points after cutting the 15th wafer. It can be seen from the two figures that a large amount of removed material powder adheres to the wire. The large-size abrasive particle surfaces in Figure 7d has been flat, indicating that the whole surface is involved in cutting. Traces of abrasive particles falling off can also be seen, but there are few pits, indicating that there are not many abrasive particles falling off. It can be seen from Figure 7e that that even in the later stage of processing, some small abrasive particles on the wire saw are still not involved in processing, and this kind of abrasive particles are generally near the position with dense abrasive particles.

**Figure 8:**
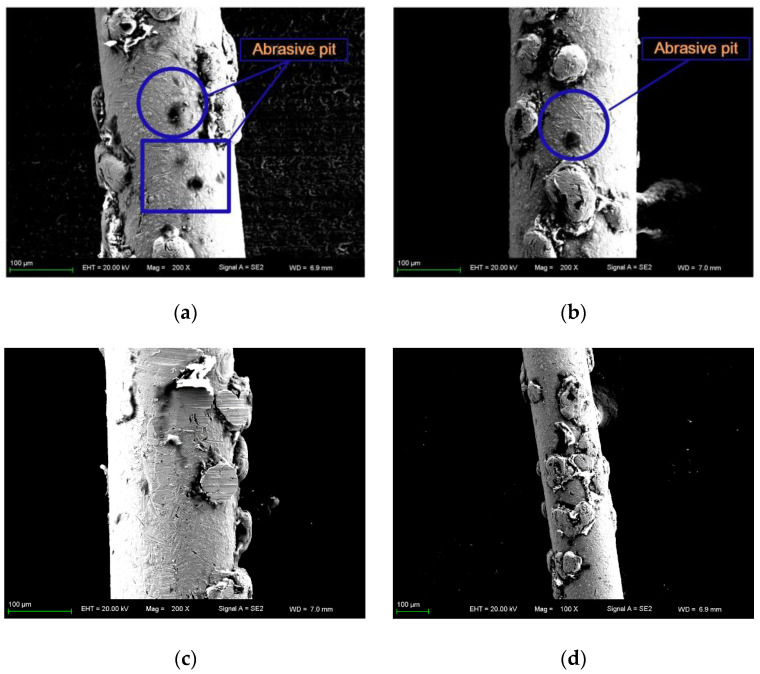
SEM image of worn wire saw. (**a**–**c**) Zoom in 200×. (**d**) Zoom in 100×.

The wire saw cannot be removed from the machine tool during processing, and the worn wire saw after wire breakage shall be observed by SEM in Figure 8. In Figure 8a,b, more pit marks of abrasive particles can be observed, and the number of dropped abrasive particles should be more than the pit marks observed because some pit marks are shallow and small and cannot be observed. Moreover, the cut wire saw surface is attached by silicon powder, especially near the abrasive particles, as shown in Figure 8d, so it also covers part of the abrasive particles falling off traces. In Figure 8c, some abrasive particles have been ground to a flat surface, and such abrasive particles have little removal ability when contacting with the part.

By observing and analyzing the wear of abrasive particles in the cutting process, it can be concluded that the wear of abrasive particles starts from the edge. With the continuation of machining, the abrasive particles surfaces are smooth. At the later stage of processing, the surfaces of some abrasive grains are smoothed, and some abrasive grains fall off. 

The macro failure mode of the saw wire is generally divided into two types, one is fatigue fracture, and the other is tensile fracture. The experiment uses reciprocating cutting. A certain length of wire saw is wound on the drum. During the cutting process, the continuous forward and reverse conversion of the drum forms the reciprocating of the wire saw. The main reason for breaking is that the pre-tightening force of the saw wire is too large, or the saw wire is impacted during the processing, which has a large sudden change force. The position where the saw wire is broken usually occurs in the reversing position. The saw wire in the reversing position needs to be decelerated and reverse accelerated to complete the reversing movement of the saw wire, which is vulnerable to large impact. Generally, the abrasive grains are not completely worn, and the number of wafer chips cut is small under this sawing wire failure mode.

**Figure 9:**
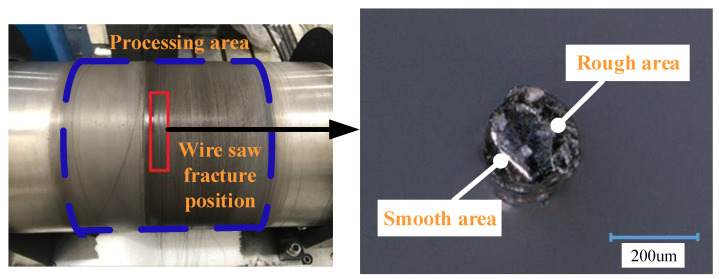
Wire saw fracture position and fracture port.

In this experiment, the wire saw breaks during wafer cutting, and the broken position is in the middle of the processing area in Figure 9. Take photos and observe the broken end of the wire saw, and there are obvious rough areas and smooth areas. It shows that the macro failure of saw wire is fatigue fracture. The reason is that under the action of long-time alternating stress, the saw wire matrix produces micro-cracks, and fatigue fracture will occur when the cracks extend to a certain extent. This failure mode is affected by the quality of the saw wire matrix. In the experiment, the abrasive particles still have the ability to remove material under the condition of fatigue fracture of the saw wire. It is important to improve the sawing quality for increasing the sawing time.

### 3.3. Surface Profile

**Figure 10:**
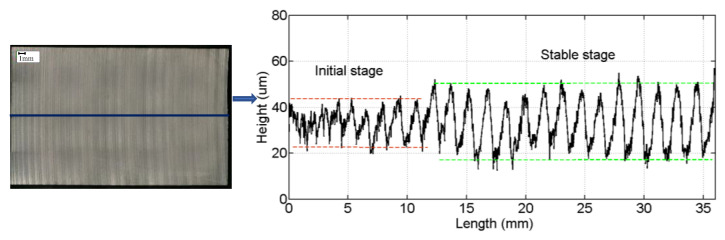
Surface profile of the wafer along the cutting direction.

The surface profile of the cut wafers is measured along the cutting direction in Figure 10. It is found that the amplitude of the profile on the wafer surface gradually increases from the beginning of cutting to the stable cutting, which is consistent with the previous cutting force research results. In a cutting process, the cutting force is divided into two stages. In the initial cutting stage, the cutting force increases from 0 N, and the depth of the single abrasive pressed into the part also increases accordingly. Therefore, the amplitude of the wafer profile curve increases continuously.

**Figure 11:**
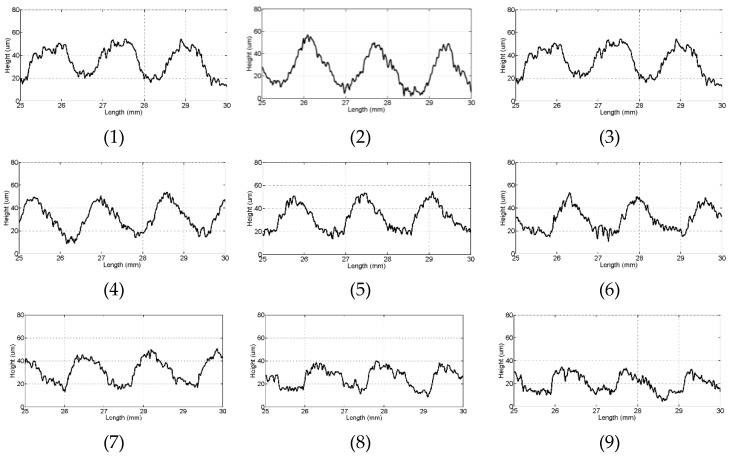

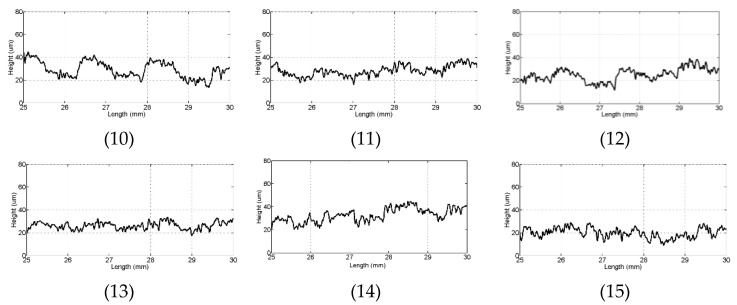
Surface profile of 15 wafers. (The number of 1–15 corresponds to the wafer number for cutting).

Figure 11 is the surface topography of the wafers from the first to the 15th cutting in the stable cutting stage. In the experiment, 15 wafers are cut, and there may be some differences in the transition stage from the initial state to the stable stage. In order to compare the surface profile of stable stage of each wafer, it is recommended to choose the area in the later cutting stage as much as possible. Each wafer enters the stable stage during the later cutting stage, so an area between 25 mm and 30 mm is selected for comparison. With the increase in cutting times, the amplitude of wafer surface profile decreases during the stable stage. From the first wafer to the tenth wafer, there are obvious peaks and valleys, and the amplitude is steadily decreasing. From the 11th wafer to the 15th wafer, the peaks and valleys of the wafer surfaces are no longer obvious. The steady decrease in the amplitude from first to tenth wafers can indicate that the abrasive particles are wearing steadily. In the process of cutting the tenth wafer, the gradual shedding of abrasive particles causes obvious changes in the surface profile when cutting the eleventh wafer, which is consistent with the state of abrasive particles when the cutting forces change explained in Section 3.1.

### 3.4. Surface Quality

The wafer surface quality after cutting has a great impact on the subsequent process. To avoid damage to the surface of the wafer, a Leica DCM3D white light confocal interference microscope is used to measure the surface roughness of the wafer. This instrument is a non-contact measurement. The test surface of the wafer is wiped clean with alcohol and it is placed on a slide. Manually the measurement point is selected. Generally, four points on a wafer are selected for measurement and averaging, and the scan area of every point is 2.41 mm × 0.95 mm. A robust Gaussian filter is used for data filtering, and the filter size is 0.08 mm × 0.08 μm and the cut-off is 0.08 mm.

**Figure 12:**
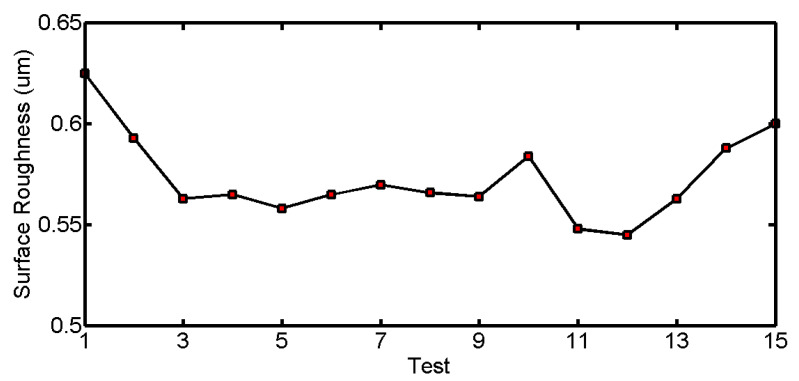
The surface roughness of the wafer changes.

Figure 12 shows the variation of the surface roughness measurement results of the wafer with the number of cuts. The vertical axis represents the value of surface roughness, and the horizontal axis represents the number of cuts. In the early stage of cutting, the surface roughness of the wafer decreases with the number of cuts (from the first wafer to the third wafer). During initial cutting, abrasive particles accumulate, and the cutting edges are many and uneven. Processing belongs to the running-in period, which is an abnormal wear stage. The rapid surface changes in contact between abrasive particles and wafers cause changes in surface roughness. During the stable wear stage, the surface roughness of the wafers is relatively stable (from the third wafer to the ninth wafer). At this stage, the abrasive particles are worn, but their shape changes are relatively small. The surface roughness of the wafers increases with the increase in cutting times in the later stage of cutting (from the 11th wafer to the 15th wafer). The general wear process includes three processes: initial wear, normal wear and sharp wear. The surface quality of the wafer during rapid wear is relatively poor. At this stage, the surfaces of the abrasive particles may be damaged, and some abrasive particles fall off during the cutting of the 10th wafer, resulting in a decrease in the surface quality of the abrasive particles. Therefore, the surface roughness of the wafer increases.

**Figure 13:**
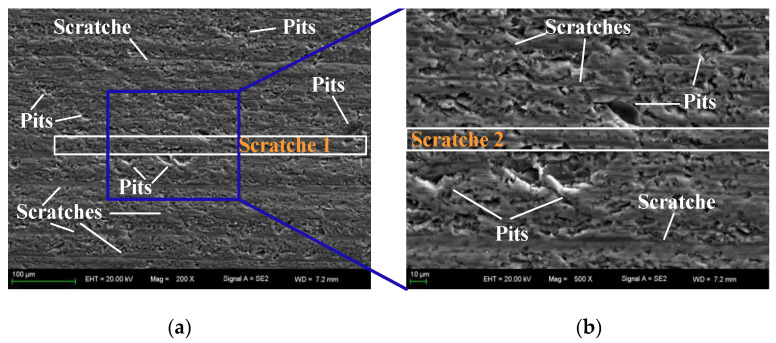

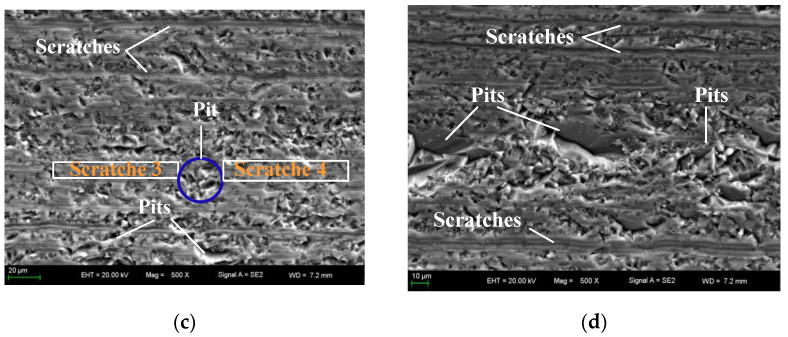
SEM photos of the first wafer (**a**) Zoom in 200×, (**b**–**d**) Zoom in 500×.

The surface of the first wafer cut was observed by SEM in Figure 13. The scratches and pits can be seen in the 200× magnified image in Figure 13a. In order to observe the pits more clearly, the surface is zoomed in to 500×, as shown in Figure 13b–d. The pits marked in Figure 13b–d belong to the surface damage. Most of the pits are caused by the collapse of the part surface material due to the interaction between large abrasive particles and the part surface. In these three photos, it can be clearly seen that in addition to the marked pits, there are also small hole-like pits, which are caused by the brittle removal of materials. The processed part is monocrystalline silicon, which has the characteristics of high hardness and brittleness. There are both brittleness removal and plasticity removal during the processing. The marks left by brittle removal material on the surface of the part are mainly pits, because the irregular crack of the material is generally distributed along the cutting direction. Generally, the abrasive particles are relatively small, and the volume of cracked material is small, which forms this kind of pit. Scratches can be seen in Figure 13d, which shows that the way of material plastic removal still exists when the abrasive particles are not worn and there are large abrasive particles at the beginning of cutting. Scratch 1 in Figure 13a and scratch 2 in Figure 13b are the same scratch. This scratch is darker than other scratches in Figure 13b, indicating that the depth of the scratch is larger, and the size of abrasive particles during plastic removal is also inconsistent. In Figure 13c, scratch 2 and scratch 3 are a continuous scratch, but there are large pits in the middle. This is because the material removal process is repeated during the cutting process. Generally, the large abrasive particles cause large pits, and the subsequent small abrasive particles plastically remove the material.

**Figure 14:**
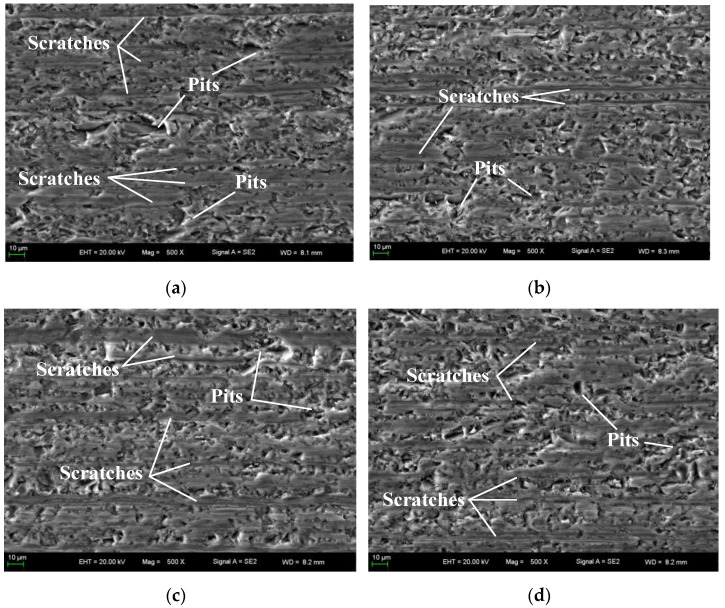

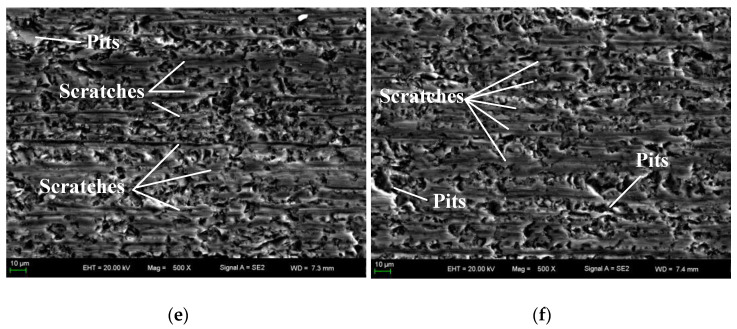
SEM photos of the cut wafer (zoom in 500×). (**a**) The fifth wafer. (**b**) The fifth wafer. (**c**) The 10th wafer. (**d**) The 10th wafer. (**e**) The 15th wafer. (**f**) The 15th wafer.

Figure 14 shows the SEM photos of the 5th, 10th, and 15th wafers after cutting at 500× magnification. There are still large pits on the fifth wafer after cutting in Figure 14a,b. Compared with the first wafer surface, the pits are much smaller overall, indicating that the number of oversized abrasive particles on the wire saw surface has been reduced in the process of cutting the fifth wafer. The distance between scratches decreases, indicating that the number of abrasive particles of plastic removal material increases at the end of superposed removal. When cutting the 10th wafer, there are fewer and fewer large pits on the surface as shown in Figure 14c,d. When cutting the 15th wafer, there are almost no large pits, and the size of the surface pits is uniform, as shown in Figure 14e,f. With the wear and fall off of abrasive particles, the large pits on the wafer surface are fewer and fewer, but there are still abrasive brittleness removal materials at the later stage of cutting.

## 4. Conclusions

Abrasive wear always exists in the cutting process of fixed-abrasive diamond wire saws. Ensuring that other processing parameters such as part feed rate, wire saw velocity, and part size remain unchanged means that the wear of abrasive particles becomes a variable. During the process of cutting single-crystal silicon with a wire saw from the new wire to a broken wire, the cutting force is measured in real-time and the cutting surface is observed. It can be seen from the experimental results that wear has an impact on the cutting force and wafer surface quality.

(1) The cutting force decreases with the increase in cutting times in the stable cutting stage, and the cutting force fluctuates in the early and later stage of machining. There are uneven, large abrasive particles in the early stage of processing, which is in the early stage of wear and running-in. In the later stage of processing, besides abrasive wear, there is also a small amount of abrasive shedding, resulting in fluctuations in cutting force.
(2) The macro failure mode of the wire saw is fatigue fractures. When the wire saw is broken, the abrasive particles on the surface of the wire saw have cutting ability, which indicates that the quality of the saw wire matrix limits the processing of the wire saw.
(3) The wear of abrasive particles starts from the edge. The wear mode of abrasive particles is mainly flattening, with fewer falling off. The difference between the peaks and troughs on the wafer surface is decreasing with the wear of abrasive particles, and when abrasive particles fall off, the reduction in surface profile fluctuations on the wafer is more pronounced. The wafer surface roughness is relatively stable during the stable wear stage and is relatively high in the early and later stages of wear. The difference in wafer surface roughness indicates that the uniformity and surface quality of abrasive particles have great impact on wafer surface quality.

Through experimental research, such phenomena have been found and summarized. However, in order to further apply the results of abrasive wear to machining, it is necessary to establish a numerical model for abrasive wear in the cutting processing and combine it with the prediction of wafer surface quality. This plays an important role in the screening and classification of wafers during actual processing.

## Figures and Tables

**Table 1:** Wire saw cutting parameters.

Title	Parameter
Part	Silicon monocrystal
Part size	36 mm × 23 mm× 200 mm
Cutting surface dimensions	36 mm × 23 mm
Part feed rate	0.75 mm/min
Wire saw velocity	1 m/s

## Data Availability

All data generated or analyzed during this study are included in this published article.
